# Editorial: Recent advances in gene modified immune cells and oncolytic virus for cancer immunotherapy

**DOI:** 10.3389/fimmu.2024.1454183

**Published:** 2024-07-04

**Authors:** Cunzhong Yuan, Yao Wang, Zong Sheng Guo

**Affiliations:** ^1^ Gynecologic Oncology Key Laboratory of Shandong Province, Qilu Hospital of Shandong University, Jinan, Shandong, China; ^2^ Department of Bio-therapeutic, the First Medical Center, Chinese PLA General Hospital, Beijing, China; ^3^ Department of Immunology, Roswell Park Comprehensive Cancer Center, Buffalo, NY, United States

**Keywords:** immune cells, oncolytic virus, genetic modification, combination, therapy, efficacy

Immunotherapy has made the most impressive process of the last few decades in the field of cancer therapeutics, as marked with the Noble Prize awarded to cancer immunotherapy in 2018 (1, 2). However, despite the impressive progress, majority of cancer patients are still resistant to CTAL4 and PD-1/PD-L1 immune checkpoint blockade (ICB)-mediated therapies. Therefore, more and innovative approaches are being developed to improve the efficacy and safety. Some key and exciting areas include either active immunotherapies such as cancer vaccines, oncolytic virus (OV)-medicated immunotherapy (3), or passive immunotherapies such as adoptive transfer of CAR T cells, TCR T cells as well as isolated, expanded and possibly gene modified tumor-infiltrating lymphocytes (TILs), in addition to novel ICB-mediated monotherapies and their combinations (4, 5).

We have selected articles in this Research Topic to illustrate recent advances in cancer immunotherapy using gene modified immune cells and OVs. The seven articles published in the Topic, with four of them original research papers and the others as reviews, include four on gene modified immune cells and four on OV-mediated therapy. In three original research, investigators have explored the combination approaches to enhance the efficacy and overcome resistance.

This Research Topic collected four studies that explored combination approaches using OV or provide cutting-edge reviews on OV-mediated immunotherapy. The review by Chen et al. have summarized the mechanism of action by OVs, limitations of monotherapy, and the potential of combinatorial approaches, especially with ICB to increase efficacy. They have discussed some hurdles and optimization approaches to further improve this promising approach of therapy. The review by Mirbahari et al. focused on oncolytic vaccinia virus and its combinations with other conventional treatment modalities to enhance efficacy. They conducted a comprehensive analysis of relevant preclinical and clinical studies, and analyzed tumor regression rates, overall survival benefits, and long-term responses. In addition, the authors provided some insights into the challenges and limitations on this specific OV-based therapies, including immune evasion mechanisms and the development of resistance.

Schober et al. have conducted a preclinical immunotherapy study with TCR-transgenic T cells and YB-1-driven oncolytic adenovirus. Their data clearly indicated that the two different classes of antitumor agents displayed synergistical antitumor effects *in vitro* and superior tumor control in a xenograft tumor model of pediatric sarcoma. In a phase I clinical study, Smith et al. demonstrated that IFNβ-armed and tyrosinase related protein 1 (TYRP1) antigen-expressing oncolytic Vesicular Stomatitis Virus (VSV) can be safely administered intratumorally or intravenously in previously treated 12 patients with metastatic uveal melanoma. Although no objective responses were seen, dose-dependent immunogenicity to the antigen TYRP1 was observed. In our opinion, it seems that one strategy to improve the potential of this double armed oncolytic VSV is to combine with another agent to elicit more potent antitumor immunity.

In the second part of the Research Topic, gene modified immune cells were explored to improve therapeutic efficacy through utility of genetically engineered immune cells to modulate the immunosuppressive tumor microenvironment (TME) or improve T cell functions in solid tumors. In the first study, Myers Chen et al. took advantage of the natural ability of myeloid cells homing to the tumor and engineered them with a Chimeric Antigen Receptor (CAR)-like immune receptor (CARIR). In this intriguing molecule, the extracellular domain is derived from the natural inhibitory receptor PD-1 while the intracellular domains are derived from CD40 and/or CD3ζ. Proof-of-principle experiments showed that co-culturing human monocytic THP-1 cells expressing CARIR with PD-L1-positive cancer cells lead to upregulation of the costimulatory molecule CD86 along with proinflammatory cytokines TNF-1α and IL-1β. Moreover, CARIR expression on THP-1 cells significantly enhanced phagocytosis of multiple PD-L1-positive cancer cells *in vitro*. In 4T1 mammary tumors, infusing murine myeloid cells expressing a murine version of CARIR significantly slowed tumor growth and prolonged survival of mice. Indeed, this study demonstrated that adoptive transfer of PD-1-CARIR-engineered myeloid cells may represent a novel strategy for treating PD-L1 positive solid cancers. In the second study, Zhang et al. have explored the utility of epigenetic approach to modulate the activity of CAR T cells. HDAC11 has been shown to be an enzyme involved in the negative regulation of T cell functions. The authors designed and tested distinct short hairpin RNA sequences targeting HDAC11 to identify the most effective one. HDAC11-deficient (downregulated by shRNA) CAR-T cells displayed better cytotoxicity against prostate cancer cells *in vitro*. This effect was attributed to enhanced activation, degranulation, and cytokine release ability of these engineered CAR-T cells. The authors revealed that HDAC11 knockdown significantly enhances CAR-T cell proliferation, diminishes exhaustion markers including PD-1 and TIM3, and promotes the formation of T central memory cells. This study illustrated that downregulation of HDAC11 could improve CAR-T cell therapy. In the last article, Yan et al. review recent discovery of γδ T cells, a specialized subset of T lymphocytes, in cancer immunotherapy.

In summary, after reading the publications in the Research Topic, we would have a better understanding of OVs, their advantages, limitations and potential strategies to overcome, leading to further improved efficacy in the future (6). The three original studies on genetically modified immune cells provided us some novel thinking and strategies to modulate the immunosuppressive TME and positively regulate and sustain the activity of T cells against tumor antigens through various signaling pathways for enhanced efficacy. Finally, we get a glimpse of properties and potential of γδ T cells in cancer immunotherapy.

## Author contributions

CY: Writing – review & editing. YW: Writing – review & editing. ZG: Writing – original draft, Writing – review & editing.

## References

[1] Sharma P Allison JP. Immune checkpoint targeting in cancer therapy: toward combination strategies with curative potential. Cell. (2015) 161:205–14. doi: 10.1016/j.cell.2015.03.030 PMC5905674 25860605

[2] Guo ZS. The 2018 Nobel Prize in medicine goes to cancer immunotherapy (editorial for BMC cancer). BMC Cancer. (2018) 18:1086. doi: 10.1186/s12885-018-5020-3 30415640 PMC6231274

[3] Ma R Li Z Chiocca EA Caligiuri MA Yu J. The emerging field of oncolytic virus-based cancer immunotherapy. Trends Cancer. (2023) 9:122–39. doi: 10.1016/j.trecan.2022.10.003 PMC9877109 36402738

[4] Zhao L Cao YJ. Engineered T cell therapy for cancer in the clinic. Front Immunol. (2019) 10:2250. doi: 10.3389/fimmu.2019.02250 31681259 PMC6798078

[5] Sterner RC Sterner RM. CAR-T cell therapy: current limitations and potential strategies. Blood Cancer J. (2021) 11:69. doi: 10.1038/s41408-021-00459-7 33824268 PMC8024391

[6] Shalhout SZ Miller DM Emerick KS Kaufman HL. Therapy with oncolytic viruses: progress and challenges. Nat Rev Clin Oncol. (2023) 20:160–77. doi: 10.1038/s41571-022-00719-w 36631681

